# Global Lipidomics Reveals the Lipid Composition Heterogeneity of Extracellular Vesicles from Drug-Resistant *Leishmania*

**DOI:** 10.3390/metabo14120658

**Published:** 2024-11-25

**Authors:** Sehyeon (Erica) Kim, Ana Victoria Ibarra-Meneses, Christopher Fernandez-Prada, Tao Huan

**Affiliations:** 1 Department of Chemistry, Faculty of Science, University of British Columbia, Vancouver Campus, 2036 Main Mall, Vancouver, BC V6T 1Z1, Canada; ericakim@chem.ubc.ca; 2 Department of Pathology and Microbiology, Faculty of Veterinary Medicine, Université de Montréal, 626 CIMIA Sicotte Street, Saint-Hyacinthe, QC J2S 2M2, Canada; ana.victoria.ibarra.meneses@umontreal.ca; 3 The Research Group on Infectious Diseases in Production Animals (GREMIP), Faculty of Veterinary Medicine, Université de Montréal, Saint-Hyacinthe, QC J2S 2M2, Canada

**Keywords:** metabolomics, lipidomics, *Leishmania*, extracellular vesicles, drug resistance

## Abstract

**Background**: The rise of drug-resistant *Leishmania* strains presents a significant challenge in the treatment of Leishmaniasis, a neglected tropical disease. Extracellular vesicles (EVs) produced by these parasites have gained attention for their role in drug resistance and host–pathogen interactions. **Methods**: This study developed and applied a novel lipidomics workflow to explore the lipid profiles of EVs from three types of drug-resistant *Leishmania infatum* strains compared to a wild-type strain. EVs were isolated through ultracentrifugation, and their lipid content was extracted using a modified Matyash protocol. LC-MS analysis was performed, and data processing in MS-DIAL enabled lipid identification and quantification. Statistical analysis in MetaboAnalyst revealed strain-specific lipid alterations, highlighting potential links between lipid composition and drug resistance mechanisms. **Results**: Our results show distinct alterations in lipid composition associated with drug resistance. Specifically, drug-resistant strains exhibited reduced levels of phosphatidylcholine (PC) and phosphatidylglycerol (PG), particularly in the amphotericin B-resistant strain *Li*AmB1000.1. Sterol and glycerolipid species, including cholesteryl ester (CE) and triacylglycerol (TG) were also found to be diminished in *Li*AmB1000.1. These changes suggest significant lipid remodeling under drug pressure, potentially altering the biophysical properties of EV membranes and their capacity for molecule transfer. Furthermore, the lipidomic profiles of EVs from the other resistant strains, LiSb2000.1 and LiMF200.5, also displayed unique alterations, underscoring strain-specific adaptations to different drug resistance mechanisms. **Conclusions**: These significant alterations in lipid composition suggest potential lipid-based mechanisms underlying drug resistance in *Leishmania*, providing new avenues for therapeutic intervention.

## 1. Introduction

Leishmaniasis is a neglected tropical disease caused by various species of the genus *Leishmania*, affecting approximately 98 countries worldwide. This disease can manifest in different clinical forms, ranging from cutaneous leishmaniasis to visceral leishmaniasis, the most severe form, which can be fatal if left untreated [1]. In the absence of vaccines against human leishmaniasis, treatment is the primary control strategy, with antimonial derivatives, amphotericin B, and miltefosine being the most commonly used drugs [2,3]. Antimony (Sb) compounds, particularly pentavalent antimonials like sodium stibogluconate, have long been the first-line treatment. Sb compounds impair macromolecule synthesis (DNA, RNA, and proteins) through the inhibition of several metabolic pathways, but resistance to these compounds has become increasingly common, particularly in regions with high treatment pressure. As an alternative, amphotericin B (AmB) is used, especially in cases of antimony resistance. AmB targets the plasma membrane of the parasite, disrupting membrane integrity. Liposomal Amphotericin B, with its enhanced tissue distribution, longer half-life, and reduced toxicity, is preferred for treating visceral leishmaniasis. Another important alternative is miltefosine (MF). It is an oral drug effective against both cutaneous and visceral leishmaniasis. MF functions by inhibiting intracellular signaling pathways and the plasma membrane.

However, the emergence of resistance to the above-mentioned antileishmanial drugs has posed a significant challenge to disease control, with relapse rates reaching up to 70% in endemic regions [4,5]. Antimonials have been the cornerstone treatment for visceral leishmaniasis (VL) in endemic regions for the past ten decades [6]. However, relapse rates range from 12% to 65% at the end of treatment and from 6% to 11% six months post-treatment [5,7,8]. Due to the significant side effects and high treatment failure rates, antimonials have been replaced as a first-line therapy [9]. Since 2013, liposomal amphotericin B has been recommended as the first-line antileishmanial drug in some VL-endemic areas due to its good efficacy and safety profile [10]. Nevertheless, its relapse rate ranges from 2.4% to 89% [11,12,13]. Miltefosine, the first effective oral drug for VL treatment, has made treatment more accessible in remote areas. However, while its cure rate is reported to be 90–95%, the relapse rate has steadily increased over the years, ranging from 6.8% to 20% [14,15,16,17]. Recent studies have revealed that extracellular vesicles (EVs) play a crucial role in the transfer of resistance to antileishmanial drugs, as well as in the interaction between the parasite and the host [18,19]. Understanding the lipid composition of *Leishmania* EVs is essential in elucidating how these components influence parasite behavior and their ability to modulate the host’s immune response. It could also potentially reveal how the parasite evades the host immune system and creates a favorable environment for its survival (i.e., inside the macrophages) [20,21,22,23]. Moreover, changes in EV lipid composition might influence the transfer of drug-resistance factors, contributing to rising drug resistance rates. This knowledge could uncover new mechanisms of pathogenesis and resistance, potentially leading to the discovery of novel antigens and immunomodulators, as well as new therapeutic targets and strategies to improve leishmaniasis treatment [19,20,21,22,23].

Lipidomics is the systematic study of small molecule lipids in a given biological system. It provides valuable insights into the biological functions of lipids. Mass spectrometry (MS) is a powerful analytical tool for analyzing lipid composition, allowing the identification and quantification of a wide array of lipid species in biological samples. Analytical strategies in lipidomics involve high-resolution MS for lipid identification, often coupled with chromatographic techniques such as liquid chromatography (LC-MS) to separate lipid species. These approaches enable comprehensive lipid profiling, offering insights into lipid-related alterations in various diseases. Previous research has employed state-of-the-art lipidomics to investigate the lipid composition of EVs derived from diverse biological sources, such as immune cells, cancer cells, adipose tissue, and blood [24,25,26].

Although lipidomics has advanced our understanding of EVs in many organisms, there remains a knowledge gap in understanding the lipid composition of *Leishmania* EVs, especially in drug-resistant strains. This work aims to fill that gap by profiling the lipids of EVs from drug-resistant and wild-type *Leishmania* strains, providing crucial insights into the lipid-based mechanisms underlying drug resistance. In more specific terms, we developed a lipidomics method to profile the lipidome of the isolated EV samples. By identifying changes in lipid composition associated with resistance, this research holds importance for the development of novel therapeutic strategies and improving the efficacy of existing treatments.

## 2. Methods and Materials

### 2.1. Leishmania Cultures

The *Leishmania infantum* wild-type strain (MHOM/MA/67/ITMAP-263) and the drug-resistant mutants *Li*Sb2000.1, *Li*AmB1000.1, and *Li*MF200.5, which were previously obtained through step-wise drug selection for resistance to Sb (potassium antimonyl tartrate, Sigma-Aldrich, Burlington, MA, USA), AmB (amphotericin B solution, Sigma-Aldrich, Burlington, MA, USA), and MF (amphotericin B solution, Sigma-Aldrich, Burlington, MA, USA), respectively [27,28,29,30,31,32], were grown in M199 medium at 25 °C in the presence of their respective EC_50_ concentrations of the drugs—2000 μM Sb, 1000 nM AmB, and 200 μM MF—to maintain drug resistance. Briefly, each resistant line was previously generated by gradually increasing the drug concentration over successive passages in vitro, starting with sublethal doses and continuing until stable resistance was achieved. The resulting mutants demonstrated resistance to the different antileishmanial drugs (Sb, MF, or AmB, respectively), as compared to the original wild-type strain. This method ensured that the selected lines acquired specific resistance traits while maintaining a common genetic background [27,28,29,30,31,32]. Of note, the drug-resistant parasites were not exposed to any drugs during the EV isolation process. The medium was supplemented with 10% fetal bovine serum, 5 μg/mL hemin, and adjusted to pH 7.0.

### 2.2. Isolation of Leishmania EVs

*Leishmania* EVs were isolated and purified following the methods we previously reported [18,20,33,34]. Briefly, *Leishmania* parasites were cultured to a density of 2.5–5.0 × 10^6^ parasites/mL in a total volume of 1 L using drug-free M199 medium at 25 °C, supplemented with 10% FBS and 5 μg/mL hemin, and adjusted to pH 7.0. The cultures were divided into ten non-ventilated 75 cm^2^ flasks (Corning, NY, USA) and maintained until they reached the late-log phase. Once the desired growth phase was achieved, the parasites were washed twice with PBS and resuspended in RPMI-1640 medium without FBS or phenol red (Life Technologies, Waltham, MA, USA) to a final concentration of 2.5–5.0 × 10^8^ parasites/mL in non-ventilated 25 cm^2^ flasks (Corning, NY, USA). The cultures were then incubated at 37 °C for 4 h to promote maximum EV release into the medium [18,33,35]. Parasite viability was assessed using propidium iodide (PI) staining before and after the 37 °C incubation, and only cultures with greater than 95% viability were used for EV purification.

Following the 4-h incubation, the samples underwent a series of centrifugation steps: first at 3000× *g* for 10 min to remove the parasites, and then at 8500× *g* for 10 min to clear cellular debris. The supernatant was subsequently filtered through 0.45 μm and 0.22 μm syringe filters. EVs were then isolated by ultracentrifugation at 100,000× *g* for 1 h using 17 mL polypropylene tubes (16 × 96 mm) in a SW 32 Ti Swinging-Bucket Rotor (Beckman Coulter, Brea, CA, USA). The isolated EVs were resuspended in an EV buffer containing 137 mM NaCl and 20 mM Hepes at pH 7.5. The protein content of the EVs was measured using the Micro BCA Protein Assay Kit (Pierce Biotechnology, Waltham, MA, USA) according to the manufacturer’s instructions. EVs were then stored in aliquots in EV buffer at −80 °C for subsequent experiments. Aliquots were carefully thawed on ice prior to use, ensuring that the EVs were never refrozen. For each strain, five separate purifications derived from five independent biological replicates were conducted.

### 2.3. EVs Visualisation Using TEM

Transmission electron microscopy (TEM) of *Leishmania* EVs was conducted according to our previously established protocol [33]. Briefly, purified EVs were placed onto formvar-coated carbon grids, then fixed for 1 min with 2.5% glutaraldehyde in 0.1 M sodium cacodylate buffer. They were subsequently stained for 1 min with 1% uranyl acetate. The grids were imaged using an HT7700 transmission electron microscope (Hitachi High Technologies Canada, Inc., Toronto, ON, M9W 6A4, Canada) operating at 80 kV.

### 2.4. EVs Size and Concentration Measurements

The size and concentration of the purified EV samples were measured using a ZetaView Nanoparticle Tracking Analyzer (NTA) (Particle Metrix, Ammersee, Germany). The analyses were conducted at 25 °C, with 0.22 μm filtered EV buffer used as the diluent. For video acquisition, the shutter frame rate was set to 45, and the sensitivity was adjusted to 85, following the software’s guidance algorithms. Following the manufacturer’s instructions, the ZetaView NTA was calibrated with 100 nm standard beads before the measurements were taken. EV samples were diluted in EV buffer with a dilution factor ranging from 1:1000 to 1:10,000 to maintain a particle count between 1000 and 2000 [18,33].

### 2.5. Lipid Extraction

Five replicates of EVs from each parasite strain, with concentrations ranging from 2 × 10^10^ to 2.4 × 10^11^ particles/mL, were processed using a modified Matyash extraction protocol. Initially, 320 μL of ice-cold methanol was added to 80 μL of EVs suspended in buffer. The EVs then underwent three freeze-thaw cycles, each consisting of 1 min in liquid nitrogen followed by 15 min in an ice-cold ultrasonic bath. Afterward, the samples were stored at −20 °C for 4 h to facilitate protein precipitation. Subsequently, 900 μL of MTBE was added, and the samples were placed on a Fisher Scientific microplate shaker for 20 min. Phase separation was induced by adding 170 μL of water, followed by centrifugation at 4 °C for 10 min at 14,000 rpm. The lipid layer was carefully transferred and dried using a CentriVap Concentrator (Labconco, Kansas City, MO, USA). Finally, the dried lipid extract was reconstituted in 80 μL ACN/IPA (1:1, *v*/*v*) for LC-MS analysis.

### 2.6. Liquid Chromatography-Mass Spectrometry Analysis

Liquid chromatography-mass spectrometry (LC-MS) analysis was conducted using an Impact II ultra-high resolution Qq-time-of-flight (UHR-QqTOF) mass spectrometer (Bruker Daltonics, Bremen, Germany) coupled with a 1290 Infinity II UHPLC system (Agilent Technologies, Palo Alto, CA, USA). Reverse-phase separation was achieved using a Waters Acquity UPLC BEH C18 column (100 mm × 1 mm, 1.7 μm) in both ESI positive and negative modes. A method blank was prepared by processing an empty vial following the same procedure as the samples. Aliquots from each sample were pooled to create a quality control (QC) sample. For the ESI positive mode, a 4 μL injection volume was used, with mobile phase A consisting of H_2_O/ACN (4:6, *v*/*v*) containing 2 mM NH_4_FA and adjusted to pH 4.8 with 0.1% formic acid. In the negative mode, an 8 μL injection volume was applied, with mobile phase A comprising H_2_O/ACN (4:6, *v*/*v*), containing 5 mM NH_4_FA, and adjusted to pH 9.8 with ammonium hydroxide. In both modes, the mobile phase B was IPA/ACN (9:1, *v*/*v*). The column was run using a gradient of A and B solutions over 35 min at a flow rate of 0.1mL/min with a sodium formate (250 mM) injection at the end of each run for mass calibration. The LC gradient was as follows: 0 min, 5% MP B; 8 min, 40% B; 14 min, 70% B; 20 min, 95% B; 23 min, 95% B. The column was equilibrated for 12 min between each injection.

### 2.7. Data Processing and Lipid Annotation

Raw LC-MS data were first converted from .d files to .abf files using Reifycs Analysis Base File Converter. The converted files were processed with MS-DIAL (version 4.6) for peak picking and feature alignment. Lipid identification was performed using the LipidBlast libraries embedded in MS-DIAL, which currently cover 29 classes of lipids: PC, lysoPC, plasmenyl-PC, PE, lysoPE, plasmenyl-PE, PS, SM, PA, lysoPA, PI, PG, CL, CerP, Cer-d, ST, [glycan]-Cer, CE, MG, DG, TG, MGDG, DGDG, SQDG, Ac2PIM1, Ac2PIM2, Ac3PIM2, Ac4PIM2, and LipidA-PP. The MS-DIAL parameters are as follows: MS1 tolerance, 0.01 DA; MS2 tolerance, 0.05 DA; and mass slice width, 0.05 Da. Features generated from MS-DIAL were further filtered in Microsoft Excel to remove lipids that elute after the analytical gradient. Furthermore, lipids whose maximum sample intensities are lower than three times the method blank intensities were removed. Intensities were normalized using the protein amount per sample. Data was further filtered to only include annotated features with MS/MS match. Features from ESI positive and negative mode were combined prior to statistical analyses.

### 2.8. Data Interpretation

MetaboAnalyst 6.0 was used for statistical analysis [36]. The data were preprocessed by log10 transformation and auto scaling. Principal Component Analysis (PCA) was performed to discern significantly altered lipids among the groups. Furthermore, ANOVAs and *t*-tests were performed to determine significantly altered lipid species whose p values were less than 0.05 and fold-changes higher than 1.5. Volcano plots illustrated the number of significantly altered lipids between the wild-type and each drug-resistant strain, highlighting whether these lipids were more abundant in the wild-type or the drug-resistant strains. Finally, class-specific analysis was also performed by combining the intensities of lipid species within the same class. A heatmap was generated to show the difference in lipid class abundance between the groups.

## 3. Results

### 3.1. EV Isolation and Characterization

**Figure 1:**
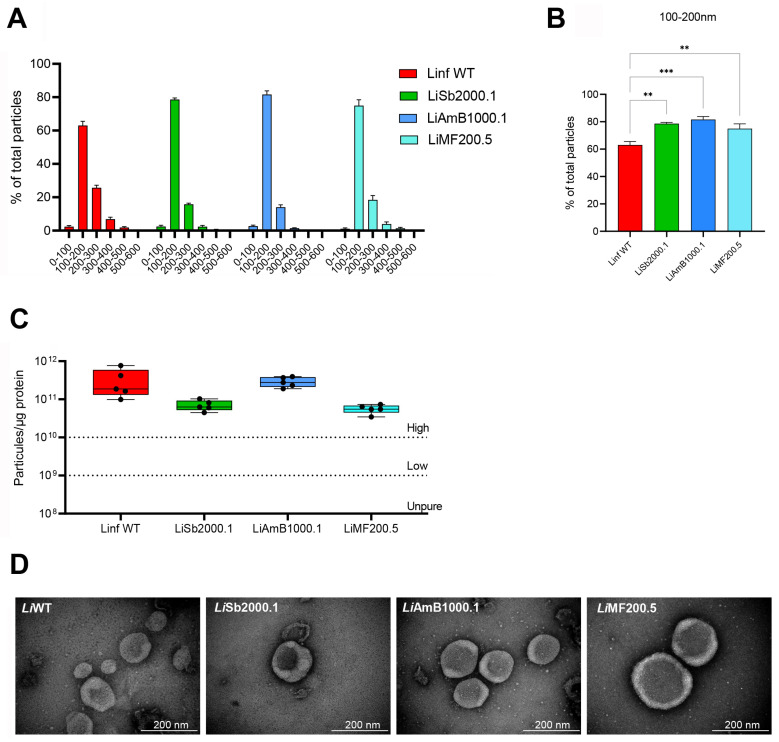
Characterization of EVs released by wild-type and drug-resistant strains of *L. infantum*. (**A**) Particle size distribution profiles were obtained via nanoparticle tracking analysis for the wild-type strain (*Li*WT) and strains resistant to antimony (*Li*Sb2000.1), miltefosine (*Li*MF200.5), and amphotericin B (*Li*AmB1000.1). (**B**) The percentage of particles corresponding to 100–200 nm. (**C**) The use of particle-to-protein ratio to quantify vesicle purity. (**D**) EVs derived from promastigotes were prepared for TEM by negative staining. Differences were statistically evaluated by unpaired *t*-test (** *p* ≤ 0.01, *** *p* ≤ 0.001).

We recently discovered that EVs released by drug-resistant *Leishmania* parasites have a distinct protein composition and can transfer functional DNA [18,35]. Building on these findings, we hypothesize that drug-resistant parasites may exhibit unique EV-lipid profiles that are associated with their resistance mechanisms. To prove this, we selected a panel of drug-sensitive and drug-resistant strains belonging to *L. infantum*. EVs were purified and evaluated for size distribution (Figure 1A) and particle-to-protein ratio (Figure 1B). Across all strains, the purified vesicles were predominantly under 200 nm in size (Figure 1A), classifying them as small EVs. The resistant strains (*Li*Sb2000.1, *Li*AmB1000.1, and *Li*MF200.5) showed very similar distributions, having a significant major proportion of vesicles in the 100-200 nm size range when compared with the WT (Figure 1B). The particle-to-protein ratio exceeded 10^10^ particles per microgram of protein for all preparations, indicating a high purity of EVs [37]. No significant differences were observed among the strains (Figure 1C). EVs were also evaluated by transmission electron microscopy (Figure 1D), confirming the predominant presence of lipid bilayer-enclosed nano-sized structures compatible with exosomes (~50 to 200 nm in diameter) and other small EVs.

### 3.2. Untargeted EV Lipidome Profiling

**Figure 2:**
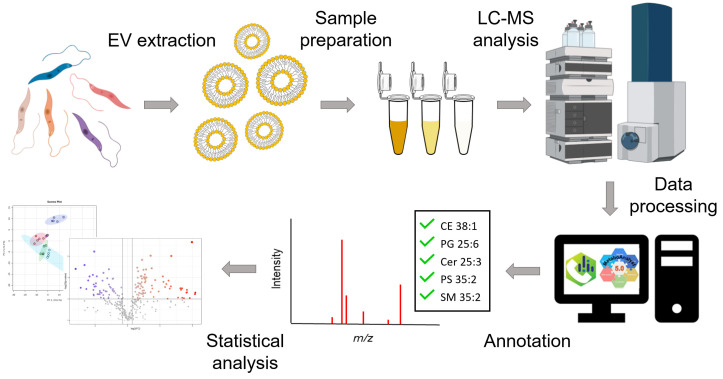
Schematic illustration of the mass spectrometry-based lipidomics workflow.

Figure 2 shows the schematic workflow for lipidomics analysis of the EV samples. In brief, EVs were collected in five independent biological replicates from *Leishmania* parasites, including wild-type, *Li*Sb1000.1, *Li*AmB2000.1, and *Li*MF200.5. Lipids were extracted from EVs and analyzed in LC-MS. Collected data were processed by MS-DIAL for peak alignment and annotations. Lastly, both univariate and multivariate statistical analyses such as *t*-tests, post-hoc ANOVA tests, and Principal Component Analyses (PCAs) were performed on the data.

**Figure 3:**
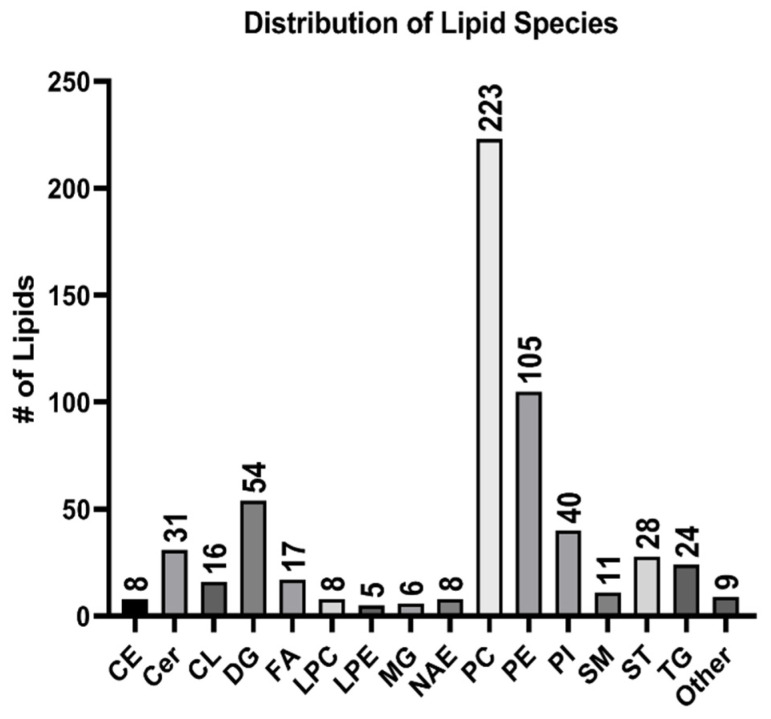
Lipid classes and their corresponding number of species detected in the EV samples.

In ESI positive mode, 1870 putative lipid species were detected in total, with 393 lipids that were confirmed via MS/MS matches. Lipid classes such as CE, DG, LPC, MG, and TG were found exclusively in positive mode. Likewise, in negative mode, 1046 putative lipid species were detected in total, with 200 lipids that have MS/MS matches. Cer, DGGA, FA, FAHFA, NAE, PEtOH, SHexCer, and SM were found only in negative mode. Notably, DG, LPE, PC, PE, PG, PI, PI-Cer, SM, and ST were present in both modes. Merged data are presented in Figure 3. Lipid classes with less than five species found, such as DGGA, FAHFA, PEtOH, PG, and SHexCer, were classified as other.

### 3.3. EV Lipidomic Comparison Between Different Types

**Figure 4:**
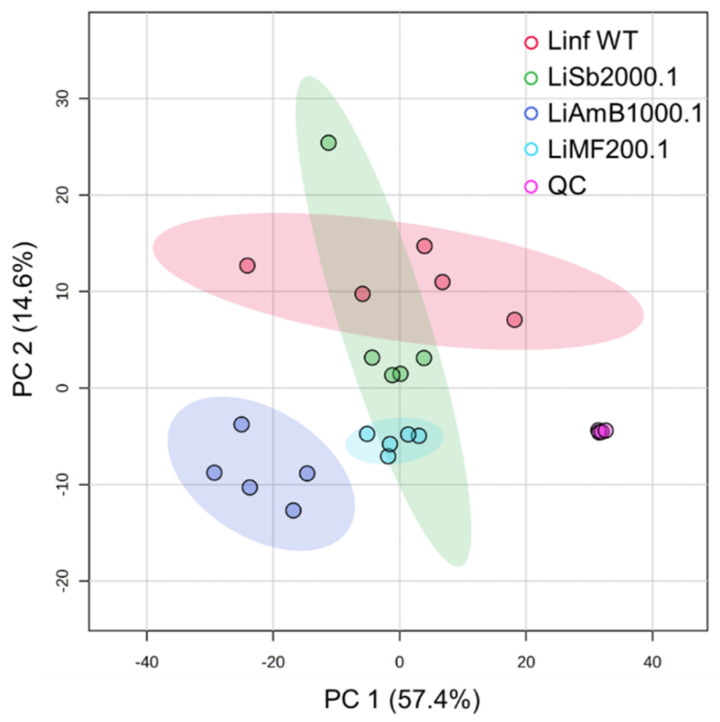
Principal component analysis of the four types of EV and quality control samples.

We then performed a quantitative comparison of the lipidomic results from the four conditions. As shown in Figure 4, *Li*AmB1000.1 exhibits the most significant divergence from the wild-type, as indicated by its greater separation against the wild-type, as well as against the other drug-resistant strains. *Li*MF200.5 also displayed distinct separation from the wild-type, albeit to a lesser extent than *Li*AmB1000.1. In contrast, *Li*Sb2000.1 overlapped with the wild-type, likely due to the substantial variation observed between one of the replicates and the others. Between the drug-resistant strains, *Li*Sb2000.1 shows an overlap with *Li*MF200.1, whereas *Li*AmB200.1 remains separate.

**Figure 5:**
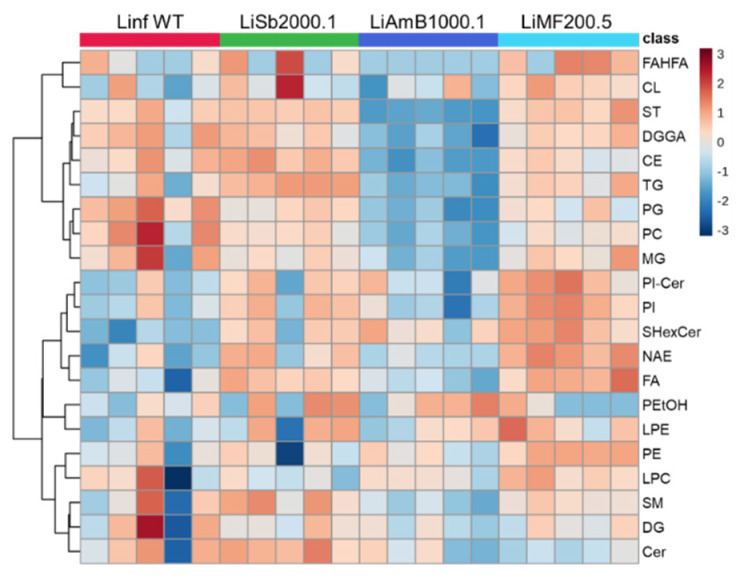
Heatmap of lipid abundance at the class level for the four EV samples. The statistical results from a post-hoc ANOVA test on lipid classes were used to construct a heatmap that combined data from both the positive and negative modes. When lipid classes were present in both modes, the mode with a higher number of species was selected. The color scale indicates the lipids’ abundance from dark blue (low) to dark red (high).

A post-hoc ANOVA test was then conducted to identify which lipid classes or species showed significant differences among the groups. In brief, ST, CE, PG, TG, DGGA, FA, PC, NAE, SHexCer, PI, and MG yielded *p*-values below 0.05, listed in the order of low to high *p*-value. Drug-resistant *Leishmania* strains exhibited lower levels of PC and PG compared to the wild-type strain, with *Li*AmB1000.1 showing the lowest abundance of these lipids. In contrast, drug-resistant strains exhibited higher abundance of SHexCer than the wild-type. Some drug-resistant *Leishmania* strains, specifically *Li*Sb2000.1 and *Li*MF200.5, showed higher levels of FA, NAE, and PI compared to the wild-type strain. Interestingly, the FA, NAE, and PI levels in the wild-type strain were comparable to those observed in the *Li*AmB1000.1 strain. *Li*AmB1000.1 displayed lower abundance of DGGA in comparison to the other strains, where abundance in the wild-type strain was comparable to that of *Li*Sb2000.1 and *Li*MF200.5. For other lipid classes such as ST, CE, and TG, *Li*AmB1000.1 also had the lowest levels, while the wild-type strain ranked second or third in abundance. In comparison, *Li*Sb2000.1 showed highest abundance among the group for CE and TG but not for ST, as *Li*MF200.5 showed the highest. A total of 88% (196/223) of the PCs, 100% (4/4) of the PGs, 100% (1/1) of the SHexCers, 71% (12/17) of the FAs, 100% (8/8) of the NAEs, 78% (14/18) of the PIs, 100% (2/2) of the DGGAs, 93% (26/28) of the STs, 100% (8/8) of the CEs, and 96% (23/24) of the TGs were significantly altered. Furthermore, the top five individual lipid species that showed most notable difference among the groups include ST 28:2, ST 28:4, ST 28:3, PC 44:11, and PC 42:9, listed in the order of low to high post-hoc ANOVA *p*-value (<0.05). Overall, *Li*AmB1000.1 displayed the lowest abundance across all five species; however, no peak was detected for ST 28:3 in both *Li*AmB1000.1 and *Li*MF200.5. Wild-type showed the second or third highest abundance in ST 28:2 and ST 28:4, and highest abundance in PC 44:11 and PC 42:9. Lastly, for ST 28:3, wild-type displayed similar abundance as *Li*Sb2000.1, which are both higher than the other two drug-resistant strains. Overall, these findings align with the patterns observed in the lipid classes, where the wild-type strain exhibited higher abundance of PC and a relatively lower abundance of ST (Figure 5).

A heatmap was constructed using *p*-values from the ANOVA test to visualize the contributions of the lipid classes to the differences between strains. Lipid classes with *p*-values less than 0.05 were also included in the heatmap for a more holistic view of the lipidome difference. Notably, the wild-type strain showed higher levels of PC and PG, while it expressed lower levels of FA, NAE, and LPE. On the other hand, LiSb2000.1 expressed a higher level of Cer but lower level of LPC. Next, LiAmB1000.1 showed lowest abundance of FAHFA, ST, DGGA, CE, TG, PG, PC, and MG as noted by the aforementioned ANOVA test results. Lastly, LiMF200.5 displayed higher levels of PI-Cer, PI, SHexCer, NAE, and FA, but lower level of Cer. Overall, there is a clear distinction in the lipid abundance patterns between the wild-type and the drug-resistant strains.

A more detailed comparison was performed using *t*-tests between the wild-type strain and the three drug-resistant strains. The *t*-tests between the wild-type and *Li*Sb2000.1 showed significant differences in SHexCer, FA, TG, and PG abundance (*p* < 0.05). Similarly, individual lipid species such as FA 44:5, FA 42:5, PE 44:8, FA 18:0, and PC 42:9 showed significant differences with wild-type displaying a lower abundance for all species except PC 42:9. When comparing wild-type to *Li*AmB1000.1, lipid classes ST, PG, CE, DGGA, PC, and SHexCer showed significant differences (*p* < 0.05) where the wild-type displayed higher abundance than *Li*AmB1000.1. Individual species such as ST 28:2, ST 28:3, ST 27:0, PC 35:6, and ST 28:4 showed the most difference (*p* < 0.05), with wild-type exhibiting higher abundance than *Li*AmB1000.1. Lastly, against the wild-type, *Li*MF200.5 showed significantly higher abundance of SHexCer, NAE, PI, PI-Cer, and FA. ST 28:2, PE P-40:6, FA 44:5, FA 42:5, and PC O-36:3 showed the most difference. With the exception of ST 28:2, *Li*MF200.5 showed higher abundance than the wild-type. Overall, when comparing the wild-type to *Li*Sb2000.1, 100% of the (1/1) SHexCer, 41% (7/17) of the FA, 58% (14/24) of the TG, and 50% (2/4) of the PG species were found to be significantly different. For *Li*AmB1000.1, 86% (24/28) of the ST, 100% (4/4) of the PG, 100% (8/8) of the CE, 100% (2/2) of the DGGA, 71% (158/223) of the PC, and 100% (1/1) of the SHexCer species were significantly different. Lastly, 100% (1/1) of the SHexCer, 88% (7/8) of the NAE, 78% (14/18) of the PI, 86% (19/22) of the PI-Cer, and 53% (9/17) of the FA species showed significant difference for *Li*MF200.5.

**Figure 6:**
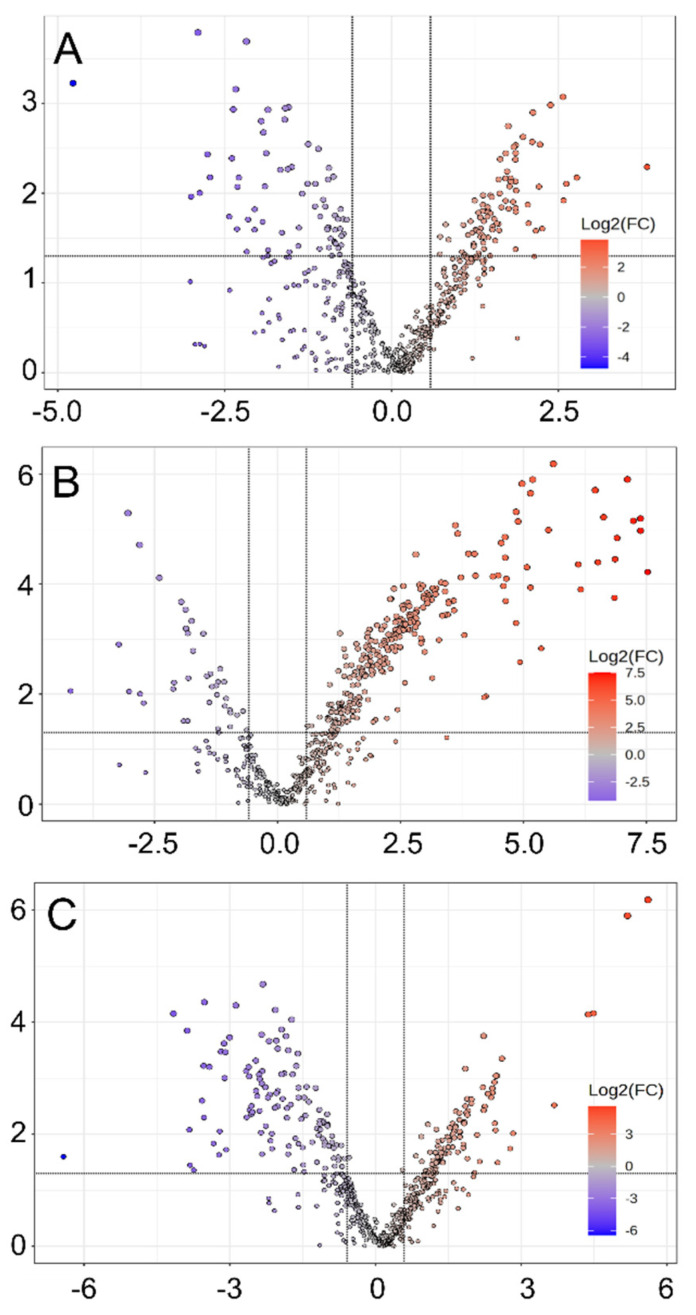
Volcano plots comparing drug-resistant strains against the wild-type. (**A**) Comparison between wild-type and LiSb1000.1. (**B**) Comparison between wild-type and LiAmB2000.1. (**C**) Comparison between wild-type and LiMF200.5. Red indicates higher fold change in the wild-type strain, while blue indicates a lower fold change in the wild-type strain.

The statistical results from the *t*-tests on the lipid species were utilized along with fold-change values to create volcano plots between the wild-type and drug-resistant strains (Figure 6). Lipid species with *p*-values less than 0.05 and fold-change values greater than 1.5 (or less than 0.67) were considered significantly changed. When comparing the wild-type to *Li*Sb1000.1, 85 lipid species were significantly higher in wild-type while 70 were significantly lower. Listed in the order of decreasing fold-change values, some of the species that showed higher abundance in wild-type include NAE 22:0, PC 40:8, and PE 40:8, while PE 44:8, SHexCer 38:1, and FA 44:5 showed lower abundance. Likewise, 272 were significantly higher and 38 were significantly lower than *Li*AmB2000.1. For instance, ST 28:4, ST 28:3, and PC 35:6 exhibited higher abundance and DG 25:4, PE O-40:6, and PE P-40:6 exhibited lower abundance. When compared against *Li*MF200.5, 96 were significantly higher and 117 were significantly lower in the wild-type. Lipid species such as ST 28:2, ST 28:3, and PC 35:6 showed higher abundance and PE 44:8, SHexCer 38:1, and PC O-41:8 showed lower.

## 4. Discussion

This study provides crucial insights into the heterogeneity of the lipid profiles of EVs released by drug-resistant *Leishmania infantum* strains and how these profiles differ from the wild-type strain. Our findings reveal significant alterations in the lipid composition of EVs from drug-resistant strains, which may contribute to their mechanisms of drug resistance. These results add to the growing body of literature highlighting the role of EVs in parasite biology, particularly in the context of drug resistance [6,7].

First of all, our untargeted lipidomics confirmed the existence of a broad range of lipids in the EV samples, including glycerolipids, glycerophospholipids, sphingolipids, sterols, and fatty acyls. These lipid classes are commonly found in EVs derived from parasites [18,19,21]. EVs from various parasitic organisms exhibit a diverse lipid profile that is essential for multiple biological functions, including cell communication, immune evasion, and modulation of host responses [22]. Glycerolipids, which encompass a range of lipid species such as triacylglycerols and diglycerides, are particularly significant in the context of EVs. They serve not only as energy reserves but also as structural components that influence the integrity and fluidity of the vesicle membranes. Glycerophospholipids and sphingolipids are also integral components of EV membranes, playing critical roles in determining their biophysical properties and functional capabilities. Furthermore, sterols contribute to the membrane’s fluidity and stability, enhancing the structural integrity of the vesicles. Fatty acyls serve as important precursors for the synthesis of bioactive lipid mediators, which can influence host–parasite interactions [23].

One of the key findings of this study was the reduction in PC and PG in drug-resistant strains compared to the wild-type strain. Glycerophospholipids play critical roles in membrane structure and function, and their reduced abundance suggests alterations in membrane dynamics and EV-mediated communication in drug-resistant parasites. The observed decrease in PC and PG aligns with previous studies that have shown lipid remodeling in response to drug pressure, particularly involving phospholipids essential for membrane integrity and vesicle formation [38]. Such lipid alterations could potentially modify the biophysical properties of EVs, influencing their ability to transfer molecules between the parasite and host, as well as their role in drug resistance.

The reduction of sterol and glycerolipid species, such as CE and TG, in the drug-resistant strains further highlights the profound lipidomic shifts induced by drug resistance. Sterols, including cholesterol, are important components of eukaryotic membranes, contributing to their stability and fluidity [23]. Cholesterol and its derivatives are often involved in drug resistance mechanisms, as they can modulate drug permeability and efflux processes [23]. The decrease in CE and TG may reflect an adaptive response by *Leishmania* to maintain membrane stability and alter lipid metabolism under drug pressure. This could affect EV production, content, and function, potentially leading to reduced drug susceptibility.

Moreover, the differences in lipid composition observed across the three drug-resistant strains suggest that lipid remodeling may vary depending on the drug and the resistance mechanisms involved. While the lipid profile of *Li*AmB1000.1 was most distinct from the wild-type, *Li*MF200.5 and *Li*Sb2000.1 also exhibited unique changes in their EV lipidomes. This strain-specific lipid alteration points to multiple adaptive strategies employed by the parasite to survive under different drug pressures. Future studies should focus on elucidating the precise roles of these altered lipids in the context of drug resistance and parasite survival.

In addition to identifying changes in lipid species, our study also highlights the importance of using advanced lipidomics techniques in parasitology. The combination of high-resolution mass spectrometry with multivariate statistical analyses, such as PCA, proved to be an effective approach for identifying significant lipid alterations across strains. This analytical strategy provides a robust platform for exploring lipid-mediated mechanisms of drug resistance and can be applied to a wide range of parasitic diseases.

Our findings highlight promising therapeutic opportunities through lipid-based interventions in drug-resistant *Leishmania* strains. Targeting enzymes involved in phosphatidylcholine and sterol biosynthesis could disrupt essential pathways that sustain parasite viability and contribute to drug resistance. Furthermore, modulating the lipid composition of EVs or inhibiting their release may interfere with the transfer of resistance factors between parasites, potentially reducing the spread of resistance [18,19]. This approach could also impact host–parasite interactions, offering a dual advantage in treatment [21,22,23]. These strategies, focused on lipid pathways, provide new avenues for overcoming drug resistance and enhancing leishmaniasis treatment outcomes.

Despite these promising findings, this study has several limitations. First, while the lipidomic analysis provided a comprehensive overview of EV lipid composition, it did not allow us to fully elucidate the functional roles of these lipids in drug resistance. Future studies should explore the mechanistic links between lipid alterations and drug resistance phenotypes, perhaps by using genetic and pharmacological tools to manipulate lipid biosynthetic pathways. Additionally, while we focused on EVs as a vehicle for drug resistance, other factors such as protein and nucleic acid cargo in EVs may also contribute to resistance and should be investigated in parallel. Finally, it is important to note that lipidomics data represent only a snapshot of the dynamic lipid landscape within these parasites, and temporal changes in lipid composition during the course of drug exposure may provide further insights.

## 5. Conclusions

In conclusion, this study reveals significant lipidomic alterations in the EVs of drug-resistant *Leishmania* strains, underscoring the importance of lipid remodeling in the development of drug resistance. By providing detailed insights into the specific lipid changes associated with drug-resistant strains, this research highlights the potential for targeting lipid pathways as a therapeutic strategy to combat drug-resistant leishmaniasis. Future research should focus on elucidating the precise roles these altered lipids play in drug resistance, exploring how interventions in lipid biosynthesis and EV-mediated communication could reverse resistance mechanisms. While this study demonstrates the power of lipidomics to uncover critical aspects of parasite biology, it also opens the door for developing new therapies aimed at disrupting the lipid-based processes that facilitate drug resistance. Lipid-targeted interventions, in combination with existing therapies, could offer a promising approach to mitigating the growing threat of drug-resistant parasitic infections.

## Data Availability

The raw data supporting the conclusions of this article will be made available by the authors on request.
